# A Plyometric Warm-Up Protocol Improves Running Economy in Recreational Endurance Athletes

**DOI:** 10.3389/fphys.2020.00197

**Published:** 2020-03-12

**Authors:** ChenGuang Wei, Liang Yu, Benedict Duncan, Andrew Renfree

**Affiliations:** ^1^School of Sport Science, Beijing Sport University, Beijing, China; ^2^School of Sport and Exercise Science, University of Worcester, Worcester, United Kingdom

**Keywords:** plyometric, resistance, warm-up, leg stiffness, post-activation potentiation, running economy

## Abstract

This study explored the impact of two differing warm-up protocols (involving either resistance exercises or plyometric exercises) on running economy (RE) in healthy recreationally active participants. Twelve healthy university students [three males, nine females, age 20 ± 2 years, maximal oxygen uptake (38.4 ± 6.4 ml min^–1^ kg^–1^)] who performed less than 5 h per week of endurance exercise volunteered to participant in this study. All participants completed three different warm-up protocols (control, plyometric, and resistance warm-up) in a counterbalanced crossover design with trials separated by 48 h, using a Latin-square arrangement. Dependent variables measured in this study were RE at four running velocities (7, 8, 9, and 10 km h^–1^), maximal oxygen uptake; heart rate; respiratory exchange rate; expired ventilation; perceived race readiness; rating of perceived exertion, time to exhaustion and leg stiffness. The primary finding of this study was that the plyometric warm-up improved RE compared to the control warm-up (6.2% at 7 km h^–1^, ES = 0.355, 9.1% at 8 km h^–1^, ES = 0.513, 4.5% at 9 km h^–1^, ES = 0.346, and 4.4% at 10 km h^–1^, ES = 0.463). There was no statistically significant difference in VO_2_ between control and resistance warm-up conditions at any velocity. There were also no statistically significant differences between conditions in other metabolic and pulmonary gas exchange variables; time to exhaustion; perceived race readiness and maximal oxygen uptake. However, leg stiffness increased by 20% (*P* = 0.039, ES = 0.90) following the plyometric warm-up and was correlated with the improved RE at a velocity of 8 km h^–1^ (*r* = 0.475, *P* = 0.041). No significant differences in RE were found between the control and resistance warm-up protocols. In comparison with the control warm-up protocol, an acute plyometric warm-up protocol can improve RE in healthy adults.

## Introduction

Distance running performance is determined by three major physiological variables; VO_2__max_; lactate threshold (LT) and running economy (RE) (Coyle, 1995). VO_2__max_ refers to the maximal volume of oxygen that the individual can uptake and utilize per minute, and is one of the key determinants of superior endurance running performance (Bassett and Howley, 2000). However, it is not possible to predict endurance running performance using VO_2__max_ alone because VO_2__max_ only sets the upper limits for the endurance performance (Costill et al., 1973; Nummela et al., 2006) and does not take into account the extent to which an athlete is able to utilize their maximal aerobic power. Therefore, despite having similar endurance performance abilities, runners may display wide variation in VO_2__max_ values, indicating that other factors play a major role in determining exercise performance. LT is generally defined as the absolute workload above which blood lactate levels rise exponentially during incremental exercises (Weltman, 1995). However, elite endurance runners may have similar values in the above variables ranging from 68.2 ml min^–1^ kg^–1^ to 84.1 ml min^–1^ kg^–1^ in VO_2__max_, and from 80% VO_2__max_ to 85% VO_2__max_ in LT, respectively (Sjodin and Svedenhag, 1985; Bassett and Howley, 2000; Barnes and Kilding, 2015). It is therefore evident that endurance performance is influenced by other variables. RE is defined as the energy demand for a given velocity of submaximal running, and is measured via steady-state oxygen uptake (Saunders et al., 2006; Barnes and Kilding, 2015). Previous research (Daniels and Daniels, 1992) indicated that improvements in RE may result in superior running performance due to a reduced energetic cost at submaximal intensities, even amongst athletes with similar VO_2__max_ values, suggesting that to some extent it may be possible to compensate for limitations in VO_2__max_ with superior RE capabilities.

Running economy is complex and multifactorial, and is related to biomechanical, metabolic, neuromuscular, and cardiorespiratory factors (Barnes and Kilding, 2015). One of the primary determinants of RE is leg stiffness (Arampatzis et al., 2006; Barnes and Kilding, 2015; Barnes et al., 2015). Stiffness can be defined as the resistance of an object or body to deformation and is calculated as the ratio of force to length (Blickhan, 1989). Dalleau et al. (1998) demonstrated that RE is associated with the stiffness of the propulsive leg, with greater stiffness eliciting the best RE. Additionally, Arampatzis et al. (2006) corroborated this finding by separating 28 distance runners into 3 groups according to RE and found the runners who had the highest leg stiffness displayed the best RE. The potential mechanism for these observations may be the redistribution of muscular output within the lower extremities and increased energy storage while running (Avela and Komi, 1998).

Active warm-up is one of the most commonly used warm-up techniques in endurance athletes as it can induce specific cardiovascular and metabolic changes that are beneficial to endurance running performance (Bishop, 2003). It is acknowledged that post-activation potentiation (PAP) can be induced by the pre-activation of skeletal muscles through heavy exercises, which is beneficial to performance in weightlifting, running and sprinting activities (Hodgson and Docherty, 2005). Barnes et al. (2015) explored the acute influence of a resistance intervention on RE and running performance in highly trained endurance runners by incorporating 20% body mass weighted vest strides as a part of the warm-up protocol. This intervention was found to enhance RE (6.0 ± 1.6%) and running performance, and regression analysis found that increased leg stiffness (*r* = 0.88) was one of the potential mechanisms of improved RE.

In addition to resistance exercises, numerous studies have also explored the effects of plyometric training on RE and running performance (Turner et al., 2003; Saunders et al., 2006; Bílý et al., 2017; Giovanelli et al., 2017; Marcello et al., 2017). Plyometric training utilizes the stretch-shortening cycle whereby a stretch of the muscle is immediately followed by a rapid muscle action (Rimmer and Sleivert, 2000). Such an action can be induced through a combination of eccentric and concentric exercises, and can be used to enhance the capability of muscles to produce power by exaggerating the stretch-shortening cycle. It includes various exercises such as bounding, jumping, and hopping (Lundin, 1985; Turner et al., 2003). Previous research has demonstrated that short-term plyometric training could enhance RE and running performance in elite endurance athletes. Blagrove et al. (2019) found that just six repetitions of a depth jump (a single set of plyometric training) could produce a moderate improvement (3.7%, effect size: 0.67) in RE in national standard male endurance runners, a similar magnitude of enhancement in RE to that achieved with a 6–14 weeks’ plyometric intervention. Barnes and Kilding (2015) hypothesized that changes in neuromuscular characteristics are associated with the improved RE following the plyometric intervention. Similarly, Cornu et al. (1997) and Spurrs et al. (2003) found that the improved RE is accompanied by an increase in leg stiffness, which allows muscles to store and utilize elastic energy more efficiently, resulting in less energy consumption while running. Essentially, endurance runners would be able to produce greater propulsion with the same or less energy consumption, which can improve RE and running performance.

Based on previous studies, it is suggested that the beneficial influences of resistance training and plyometric training on RE and running performance may be derived from the PAP effect and/or increased leg stiffness. However, there no study has explored the effectiveness of the two warm-up protocols on RE in healthy adult recreational athletes. Therefore, the purpose of this study is to determine whether acute resistance and plyometric warm-up protocols can improve RE in healthy adults. It was hypothesized that, compared with a control warm-up, the plyometric and resistance warm-up protocols would contribute to larger improvements in RE and leg stiffness without significant changes in other metabolic and pulmonary gas exchange indicators, RPE, perceived race readiness, time to exhaustion, or VO_2__max_.

## Materials and Methods

### Subjects

Twelve healthy university students (three males, nine females, age 20 ± 2 years; body mass 58.8 ± 8.5 kg; body height 165.8 ± 7.6 cm; BMI 21.3 ± 2.1 kg m^–2^; VO_2__max_ 38.6 ± 6.3 ml min^–1^ kg^–1^; body fat percentage 25.5 ± 6.4%) volunteered to participate in this study (Table 1). All participants were (1) free from any cardiovascular and neurological diseases, and were not suffering from any musculoskeletal injuries; (2) were not participating in systematic endurance training, and had a total exercise load of less than 5 h per week. All participants provided written informed consent prior to participation in any of the experimental procedures that had received prior ethical approval at the University of Worcester.

**TABLE 1:** Subjects characteristics (*N* = 12, 9 females, 3 males).

Characteristics	Mean ± SD
Age (year)	20.25 ± 2.3
Body mass (kg)	58.8 ± 8.5
Body height (cm)	165.8 ± 7.6
BMI (kg m^–2^)	21.3 ± 2.1
Body fat percentage (%)	25.5 ± 6.4
VO_2max_ (ml min^–1^ kg^–1^)	38.6 ± 6.3
Training time (h week^–1^)	<5

### Procedures

Participants completed three experimental sessions (control, plyometric, and resistance warm-up) in a counterbalanced crossover design, using a Latin-square arrangement, with trials separated by 48 h. An initial visit was used to familiarize participants with testing equipment and procedures. Participants were instructed to perform no strenuous exercise within the 48 h prior to testing in order to avoid fatigue and delayed muscle soreness. They were also instructed to refrain from caffeine and alcohol consumption in the 24 h prior to testing, and to avoid the consumption of food in the 3 h prior to each testing session. All the tests were conducted in a laboratory with similar temperature and humidity (21.6 ± 1.4°C, 48 ± 5%), and at the same time of the day for each subject to avoid any influences of the circadian rhythm.

In the control and resistance conditions, participants performed a 10-min self-paced jog on a motorized treadmill followed by six, 10 s strides with or without extra load with a 1-min rest period between each on treadmill. The velocity of strides during the first condition was controlled by participants, and the velocity was recorded and repeated in the next condition. In the resistance condition, subjects performed the strides whilst wearing a weighted vest equal to 20% of body mass (Barnes et al., 2015). The warm-up procedure was followed by a 10-min recovery, then followed by five maximal continuous straight-leg jumps for the determination of leg stiffness. At the end of the rest following warm-up, perceived race readiness for each subject was determined. Participants then performed the running tests on a motorized treadmill.

**FIGURE 1:**
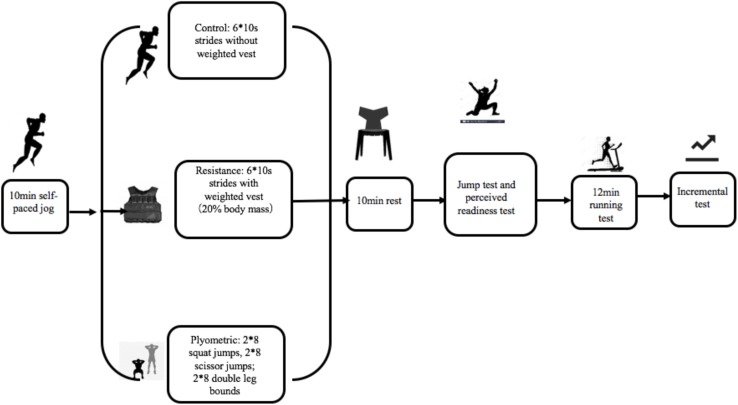
Experimental protocol.

During the plyometric intervention, subjects performed 2 × 8 squat jumps, 2 × 8 scissor jumps, and 2 × 8 double leg bounds (2 sets of 8 repetitions) as a part of warm-up, and had 60 s to recover between each set. Prior to the intervention, participants were shown the technique to be used during jumping through use of three videos. In the squat jumps, participants were required to start with feet wide and chest up, to squat low so that thighs were parallel with the ground, then drive their arms up and push off the floor. In the scissor jumps, participants were requested to stand with one leg in front and one leg behind, maintain a right angle between thigh and calf, then drive their arms up and push off the floor whilst reversing leg positions. In the double leg bounds, the participants commenced from the same starting position as in the squat jumps. However, they then jumped forward as far as possible with the arms up. The total amount of time spent in each of the three warm-up protocols was recorded. Participants were instructed to wear the same pair of running shoes during the three tests. The full experimental protocol is illustrated in Figure 1.

### Measurement

#### Anthropometric Characteristics Measurement

Body height and body mass were measured using a Seca 213 Stadiometer (Seca, United Kingdom) and Sartorius Combics scales (Bovenden, Germany). A Bodystat (Isle of Man, British Isles) device was used to measure body fat percentage through two electrodes positioned on the participants’ right hand and foot joints.

#### 5-Jump Plyometric Test

Participants conducted five maximal continuous straight-leg jumps on a force plate (Watertown, MA, United States). They were requested to keep the legs as straight as possible throughout jumping and to try to obtain the maximum height on each jump with the contact time kept as fast as possible. Leg stiffness was calculated as the relative power (N kg^–1^) divided by the vertical displacement (m) measured during the 5-jump plyometric test (Morin et al., 2005). The leg stiffness was calculated using the following formula: *K*_leg_ = *F*_max_⋅ΔL^−1^ (*F*_max_ means the maximal ground reaction force during the contact; ΔL refers to the peak displacement of the leg spring (Morin et al., 2005). The peak value was selected for the subsequent data analysis.

#### Perceived Race Readiness

At the end of rest following the three warm-up protocols, participants were inquired “how effectively do you think the warm-up was in preparation for racing?” and requested to rate their readiness from 1 (not effective at all) to 10 (extremely effective) (Ingham et al., 2013).

#### Running Test and Incremental Test

Initial velocity was 7 km h^–1^ and increased by 1 km h^–1^ every 3 min up to 10 km h^–1^. The gradient of the motorized treadmill was set at 1% to simulate the air resistance that athletes experience on an outdoor track (Jones and Doust, 1996). During the incremental test, at 10 km h^–1^, the gradient increased by 2.5% every 2 min until exhaustion. HR and pulmonary gas-exchange indicators were measured continuously with a Polar H7 heart rate monitor (Polar, United Kingdom) and Cortex Metalyzer (Cranlea, United Kingdom). The VO_2_, HR, *V*_*E*_, and RER were averaged over the last minute of each running velocity. VO_2__max_ was determined to have been achieved when two of three criteria were achieved (1) RER >1.1; (2) VO_2_ reached a plateau or decreased slowly in the final stage of the test; (3) HR attained over 90% of the age predicted maximum (maximal HR = 220 - age) (Buckley et al., 2015). RPE was obtained using the Borg Category (6–20) scale (Borg, 1998) in the final 30 s at each velocity.

## Data Analysis

Normality of data was assessed using the Shapiro–Wilk test prior to analysis. A two-way repeated measures ANOVA (analysis of variance) was used to analyze the differences in each variable within-subject factor: warm-up conditions (control, plyometric, and resistance warm-up protocols); within-subject factor: different velocities (7, 8, 9, and 10 km h^–1^). A two-way repeated measures ANOVA followed by least significant difference (LSD) *post hoc* test and simple effects analysis where appropriate, were used to analyze the pairwise comparisons. Perceived race readiness, leg stiffness, and time to exhaustion within three warm-up interventions were assessed with one-way ANOVA. The magnitude of differences in key dependent variables were presented as effect sizes using the following criteria 0.2–0.5 small; 0.5–0.8 moderate; >0.8 large (Cohen, 1988). A Pearson correlation was used to assess the relationship between changes in leg stiffness and VO_2_ (RE). Statistical analyses were performed using SPSS.24. All data is presented as Mean ± SD, and statistical significance was accepted at *P* < 0.05.

## Results

There were interaction effects between the three warm-up protocols and four running velocities for VO_2_ [*F*(6,66) = 2.365, *P* = 0.040], while there were no interaction effects for *V*_*E*_ [*F*(2.52,27.70) = 0.257, *P* = 0.823], HR [*F*(2.49,27.46) = 0.618, *P* = 0.581], RER [*F*(1.46,16.12) = 2.045, *P* = 0.169] or RPE [*F*(6,66) = 1.548, *P* = 0.215]. In addition, none of the three warm-up protocols had any main effects on *V*_*E*_ [*F*(2,22) = 0.591, *P* = 0.562], HR [*F*(2,22) = 1.723, *P* = 0.202], RER [*F*(1.247,13.715) = 0.006, *P* = 0.966] or RPE [*F*(2,22) = 1.069, *P* = 0.360].

### Effect of Running Velocity on VO_2_

VO_2_ increased with increased velocity (control warm-up protocol: *F* = (3,33) = 119.109, *P* < 0.01; Plyometric warm-up protocol: *F* = (3,33) = 60.682, *P* < 0.01; Resistance warm-up protocol: *F* = (3,33) = 241.410, *P* < 0.01). Values for VO_2_ following each warm-up protocol and at each running velocity are presented in Table 2.

**TABLE 2:** The effects of four running velocities on VO_2_ following the control, plyometric, and resistance warm-up protocols.

Interventions	7 km h^–1^	8 km h^–1^	9 km h^–1^	10 km h^–1^	*P* values within 4 velocities
Control warm-up	26.75 ± 2.667	30.08 ± 3.423**	33.58 ± 3.605**	36.08 ± 3.630**	<0.01
Plyometric warm-up	25.08 ± 2.39	27.33 ± 4.29**	32.08 ± 2.61**	34.50 ± 3.55**	<0.01
Resistance warm-up	26.00 ± 2.70	30.67 ± 3.34**	33.83 ± 3.83**	36.42 ± 3.58**	<0.01

Significant difference (**P < 0.01) from 7 km h^–1^.

### Effect of Warm-Up Protocol on VO_2_

**FIGURE 2:**
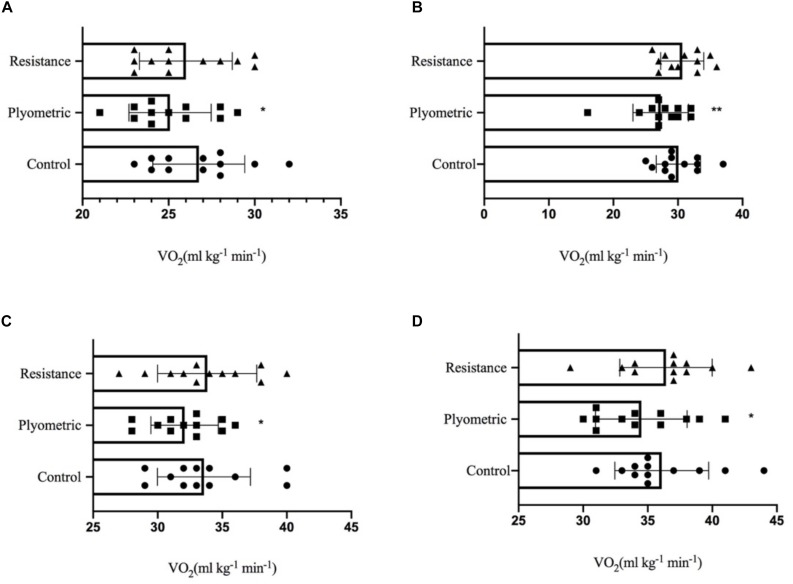
Individual values and Mean ± SD for VO_2_ at the velocity of 7 km h^– 1^ **(A)**, 8 km h^– 1^ **(B)**, 9 km h^– 1^ **(C)**, and 10 km h^– 1^ **(D)** within three warm-up protocols. Significant difference (^∗^*P* < 0.05, ^∗∗^*P* < 0.01) from control warm-up.

In comparison with the control warm-up protocol, at 7 km h^–1^, VO_2_ was lower in the plyometric condition [control: 26.75 ± 2.66 ml min^–1^ kg^–1^; plyometric: 25.08 ± 2.39 ml min^–1^ kg^–1^ (*F*(2,22) = 3.368, *P* = 0.032, ES = 0.355)]. There was no significant difference in VO_2_ between the control and resistance exercise conditions [26 ± 2.69 ml min^–1^ kg^–1^ (*F*(2,22) = 3.368, *P* = 0.202, ES = 0.144)]. Individual values and Mean ± SD for VO_2_ at the velocity of 7 km h^–1^ within the three protocols are displayed in Figure 2A.

Similarly, at all velocities, VO_2_ was significantly lower following the plyometric warm-up protocol compared to the control condition: 30.08 ± 3.42 ml min^–1^ kg^–1^ to 27.33 ± 4.30 ml min^–1^ kg^–1^, [*F*(2,22) = 8.781, *P* = 0.006, ES = 0.513] (8 km h^–1^), 33.58 ± 3.61 ml min^–1^ kg^–1^ to 32.08 ± 2.61 ml min^–1^ kg^–1^, [*F*(2,22) = 4.287, *P* = 0.034, ES = 0.346] (9 km h^–1^) and 36.08 ± 3.63 ml min^–1^ kg^–1^ to 34.50 ± 3.55 ml min^–1^ kg^–1^, [*F*(2,22) = 4.653, *P* = 0.010, ES = 0.463] (10 km h^–1^), respectively. However, no statistically significant differences were found in VO_2_ between control and resistance warm-up protocols (*P* = 0.570, ES = 0.030) at 10 km h^–1^. Individual values and Mean ± SD for VO_2_ at the velocity of 8, 9, and 10 km h^–1^ within three warm-up protocols are shown in Figures 2B–D.

### Effect of Warm-Up Protocol on Perceived Race Readiness, Leg Stiffness and Time to Exhaustion

**FIGURE 3:**
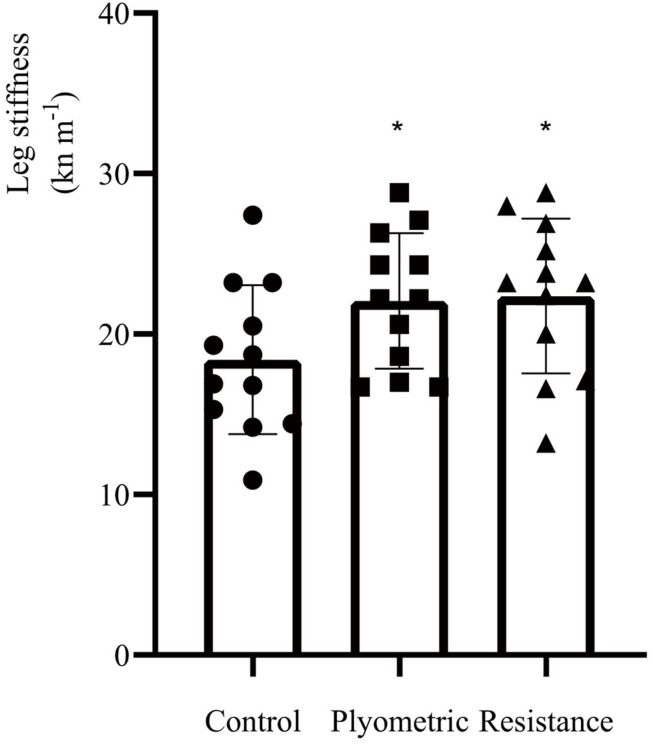
Individual values and Mean ± SD for leg stiffness within three warm-up protocols. Significant difference (^∗^*P* < 0.05) from control warm-up.

No statistical significant changes were found in perceived race readiness or time to exhaustion between the warm-up protocols. Leg stiffness showed significant increases following the plyometric and resistance warm-up protocols, increasing from 18.59 ± 4.50 kN m^–1^ to 22.38 ± 3.91 kN m^–1^, *F*(2,33) = 3.754, *P* = 0.039, ES = 0.541 and 23.08 ± 4.51 kN m^–1^, *F*(2,33) = 3.754, *P* = 0.016, ES = 0.765 (Table 3). Individual values and Mean ± SD for leg stiffness following the three warm-up protocols are shown in Figure 3.

**TABLE 3:** Influence of 3 protocols of warm-up on leg stiffness, perceived race readiness, and time to exhaustion.

Indicators	Control	Plyometric warm-up	Resistance warm-up
Leg stiffness	18.59 ± 4.50	22.38 ± 3.91*	23.08 ± 4.51*
Perceived race readiness	4.67 ± 1.37	5.08 ± 1.62	5.00 ± 2.00
Time to exhaustion	14.09 ± 2.50	14.09 ± 2.39	14.43 ± 2.60

Significant difference (*P < 0.05) from control warm-up.

### Correlations Between Changes in VO_2_ and Changes in Leg Stiffness at Each Running Velocity Following the Plyometric Warm-Up Protocol

No statistically significant correlations were found between changes in VO_2_ and changes in leg stiffness at velocities of 7 km h^–1^(*r* = 0.058, *P* = 0.185), 9 km h^–1^ (*r* = 0.226, *P* = 0.057), or 10 km h^–1^ (*r* = 0.050, *P* = 0.187) following the plyometric warm-up protocol. However, increased leg stiffness was moderately correlated with improved RE at 8 km h^–1^ (*r* = 0.475, *P* = 0.041). In addition, no statistically significant correlations were found between changes in VO_2_ and changes in leg stiffness at velocities of 7 km h^–1^ (*r* = -0.154, *P* = 0.063), 8 km h^–1^ (*r* = -0.226, *P* = 0.057), 9 km h^–1^ (*r* = -0.050, *P* = 0.187), or 10 km h^–1^ (*r* = -0.080, *P* = 0.198) following the resistance warm-up protocol.

## Discussion

This study investigated the influences of plyometric and resistance warm-up protocols on RE in healthy adults. The primary finding of this study was that a plyometric warm-up can improve RE (6.2% at the velocity of 7 km h^–1^, ES = 0.355, 9.1% at the velocity of 8 km h^–1^, ES = 0.513, 4.5% at the velocity of 9 km h^–1^, ES = 0.346, and 4.4% at the velocity of 10 km h^–1^, ES = 0.463) with no statistically significant changes in other metabolic and pulmonary gas exchange indicators, RPE, time to exhaustion, perceived race readiness or VO_2__max_ in comparison to a control protocol. However, no significant differences were found in RE at any velocity between the control and resistance protocols even though the leg stiffness showed a significant increase (24%, ES = 0.765) following the resistance warm-up intervention.

Numerous studies have explored the influence of chronic (ranging from 4 to 14 weeks) plyometric and/or resistance training interventions on RE and running performance (Spurrs et al., 2003; Guglielmo et al., 2009; Berryman et al., 2010). Balsalobre-Fernández et al. (2016) suggested it is optimal and practical for highly trained endurance athletes to perform 8–12 weeks’ low to high intensity resistance and plyometric training, with a frequency of 2–3 sessions per week, for the purpose of enhancing RE. In contrast, few studies have explored the acute effects of plyometric and resistance warm-up protocols on RE in healthy adults and endurance athletes. Even though an enhancement in RE was found in the present study following the plyometric warm-up, it is unclear whether the improvement can be translated to competitive endurance athletes using the same protocol.

In comparison with the control warm-up, leg stiffness increased by 20% (*P* = 0.039, ES = 0.541) and 24% (*P* = 0.016, ES = 0.765) following plyometric and resistance warm-up protocols, respectively. Significant correlations between changes in RE and changes in leg stiffness following the plyometric warm-up intervention were found at 8 km h^–1^ only. Previous research has found improved RE and increased leg stiffness following plyometric and resistance interventions (Millet et al., 2002; Spurrs et al., 2003; Barnes et al., 2015; Moore, 2016). However, in the present study, only two participants demonstrated improvements in RE following the resistance warm-up protocol. One possibility is that the exercise intensities utilized in the present study were inappropriate for the purpose of inducing any PAP effect. Resistance exercise can induce PAP, which may have beneficial effects on RE and running performance by increasing the phosphorylation of myosin regulatory light chains and Ca^2+^ sensitivity in striated muscles (MacIntosh, 2010; Boullosa et al., 2018). However, fatigue and PAP effect can exist in the body at the same time (Rassier and Macintosh, 2000), and is dependent not only *n* training protocols, but also the individual’s fitness level (Hamada et al., 2000). In the present study, a 20% body mass weighted vest was used, because this loading had previously been demonstrated to increase leg stiffness and improve RE in more well trained runners (Barnes et al., 2015). Given the difference in training status of the experimental participants, a possible reason why the resistance warm-up produced no change in RE in our study may be that the 20% body mass weighted vest was inappropriate for inducing a PAP effect in this population. If the load was too heavy, then the negative effects of fatigue on RE may have counteracted any beneficial influences of a PAP effect on RE and running performance. Alternatively, it may simply be the case that the protocol utilized was insufficient to produce any PAP effect in the first place. Given the data available to us, we are unable to determine if the absence of benefit of the resistance protocol resulted from inability to induce PAP, or resultant fatigue. However, it is noteworthy that excessive fatigue was not apparent during testing itself and it that there were no differences in perceived race readiness between conditions, which may be expected to be the case if the vest was indeed too heavy. Additionally, it may also suggest that leg stiffness is not the only factor of influencing RE.

Running economy was improved at all running velocities following the plyometric warm-up protocol. However, increased leg stiffness was only moderately (*r* = 0.475, *P* = 0.041) correlated with improved RE at 8 km h^–1^, and not at other velocities. This may suggest that improved leg stiffness was not the only factor responsible for the enhanced RE following the plyometric warm-up. PAP effects have previously been demonstrated to be induced through the pre-activation of skeletal muscles through heavy exercises (Hodgson and Docherty, 2005), and Blagrove et al. (2019) found that just six repetitions of a depth jump produced moderate improvements in RE in national standard male endurance runners through inducing a PAP effect. PAP effects can increase phosphorylation of myosin regulation light chains and Ca^2+^ sensitivity in striated muscle (Boullosa et al., 2018), leading to an increased rate of force development, and peak tension through an increased number of cross-bridges formed (MacIntosh, 2010). These neuromuscular adaptations may allow runners to maintain a constant running velocity with a relative low energy cost (Blagrove et al., 2018). In addition, any PAP effects induced by a plyometric warm-up may potentiate the recruitment of type I muscle fibers, thereby postponing the activation of less efficient type II muscle fibers and reducing energy consumption during running (Schumann et al., 2016). Moreover, elastic energy induced by a plyometric warm-up can be stored in the tendons and skeletal muscles, making an extensive contribution to propulsion (Anderson, 1996). This may reduce ground contact times, and is likely to further reduce energy consumption (improve the RE) during endurance exercise. The above mechanisms may explain the improved RE following the plyometric warm-up protocol. However, we acknowledge that in the present study, PAP effects were not specifically measured, meaning our ability to fully explain the underpinning mechanisms responsible for the observed effects on RE and performance is limited.

### Limitations

Limitations to the current study include (1) Total time for the three warm-up interventions were slightly different, being 16 min for the control condition, 16 min for the resistance condition and an average of 16.34 min (ranging from 15.22 to 17.3 min) for plyometric condition. (2) As described above, the possible reason of resistance warm-up inducing no change in RE in this present study may be the unsuitable intensity, leading to fatigue in participants. However, no measure of fatigue was made during the study. (3) Even though enhancement in RE was found in present study following the plyometric warm-up, it is unclear whether the improvement can be translated to the competitive endurance athletes using the same plyometric warm-up protocol. Therefore, the present study suggests that more attention should be paid to explore the optimal intensity of resistance training using weighed vest by monitoring some fatigue and PAP effect related indicators, and also explore the beneficial effects of acute plyometric warm-up on RE and running performance in elite endurance athletes.

## Conclusion

In conclusion, the primary finding of this study was plyometric warm-up can improve RE (6.2% at 7 km h^–1^, ES = 0.355, 9.1% at 8 km h^–1^, ES = 0.513, 4.5% at 9 km h^–1^, ES = 0.346 and 4.4% at 10 km h^–1^, ES = 0.463) (Supplementary Datasheet 1). However, no statistical significant changes in other metabolic and pulmonary gas exchange indicators, time to exhaustion, perceived race readiness, and VO_2__max_ were found in comparison with the control and resistance warm-up protocols. In addition to this, increased leg stiffness following the plyometric warm-up protocol was related to the improved RE at the velocity of 8 km h^–1^ (*r* = 0.475, *P* = 0.041) in healthy adults. Future studies should endeavor to elucidate the effect of plyometric warm-up protocols on RE and running performance in elite endurance runners.

## Data Availability Statement

All datasets generated for this study are available on request to the corresponding author.

## Ethics Statement

The studies involving human participants were reviewed and approved by the Ethical Committee of the University of Worcester, United Kingdom. All participants provided informed consent prior to participation in experimental procedures.

## Author Contributions

CW, BD, LY, and AR were involved in study design and data interpretation. CW collected and analyzed the data. All authors approved the final version of manuscript.

## Conflict of Interest

The authors declare that the research was conducted in the absence of any commercial or financial relationships that could be construed as a potential conflict of interest.

## Abbreviations

ANOVA: analysis of variance ES effect size
BMI: body mass index
HR: heart rate
LSD: least-significance difference
LT: lactate threshold
PAP: post-activation potentiation
RE: running economy
RER: respiratory exchange rate
RPE: rating of perceive exertion
SD: standard deviation
*V*_*E*_: expired ventilation
VO_2_: oxygen uptake
VO_2max_: maximal oxygen uptake.
